# Comparison of early osseointegration of non-thermal atmospheric plasma-functionalized/ SLActive titanium implant surfaces in beagle dogs

**DOI:** 10.3389/fbioe.2022.965248

**Published:** 2022-10-26

**Authors:** Li Long, Min Zhang, Shuaiqi Gan, Zheng Zheng, Yanjin He, Jia Xu, Ruijie Fu, Qiang Guo, Deping Yu, Wenchuan Chen

**Affiliations:** ^1^ State Key Laboratory of Oral Diseases and National Clinical Research Center for Oral Diseases, West China Hospital of Stomatology, Sichuan University, Chengdu, China; ^2^ School of Mechanical Engineering, Sichuan University, Chengdu, China

**Keywords:** non-thermal atmospheric plasma, osseointegration, hydrophilicity implant, SLActive implant, SLA implant

## Abstract

**Background:** Hydrophilic dental implants are gaining increasing interest for their ability to accelerate bone formation. However, commercially available hydrophilic implants, such as SLActive™, have some major limitations due to their time-dependent biological aging and lower cost-effectiveness. The non-thermal atmospheric plasma (NTAP) treatment is a reliable way to gain a hydrophilic surface and enhance osseointegration. However, a few studies have been carried out to compare the osseointegration of NTAP-functionalized titanium implants and commercially available hydrophilic implants.

**Purpose:** In this study, we compare the osseointegration abilities of the NTAP-functionalized titanium implant and Straumann SLActive.

**Material and methods:** The NTAP effectiveness was examined using *in vitro* cell experiments. Then, six beagle dogs were included in the *in vivo* experiment. Straumann SLActive implants, SLA implants, and SLA implants treated with NTAP were implanted in the mandibular premolar area of dogs. After 2 w, 4 w, and 8 w, the animals were sacrificed and specimens were collected. Radiographic and histological analyses were used to measure osseointegration.

**Results:** NTAP treatment accelerated the initial attachment and differentiation of MC3T3-E1 cells. In the *in vivo* experiment, bone parameters (e.g., BIC value and BV/TV) and volume of new bone of NTAP groups were close to those of the SLActive group. Additionally, although there was no statistical difference, the osseointegration of SLActive and NTAP groups was evidently superior to that of the SLA group.

**Conclusion:** NTAP-functionalized implants enhanced cell interaction with material and subsequent bone formation. The osseointegration of the NTAP-functionalized implant was comparable to that of the SLActive implant at the early osseointegration stage.

## 1 Introduction

The biofunctionality of a dental implant is strongly affected by its surface features; therefore, many methods to modify the surface of an implant have been proposed to date, of which roughness, wettability, and chemical coating have proved to be the most effective for the implantation to be successful (Le Guéhennec et al., 2007; Rupp et al., 2018). This nanometer- or micrometer-level roughness is conducive to the adhesion and proliferation of osteoblasts on the titanium implant surface, and this roughness can be achieved by the large grit and acid etching (SLA) technology (Kunrath et al., 2020). However, poor osseointegration and bacterial infection are common problems leading to the failure of implantation in a clinical procedure. Wettability or the surface energy of implants is another important factor affecting osseointegration. The surface energy of a traditional implant is relatively low due to the absorption of hydrocarbons and carbonates from the air (Rupp et al., 2018). The hydrophilic implant surface carries a higher density of hydroxyl group functionality (-OH) and amine group functionality (-NH_2_), which enhances protein absorption and allows better interaction with cells.

Chemical reactions, ultraviolet (UV) radiation exposure, and plasma and ozone exposures can activate the material surfaces with reactive groups, thus increasing the surface energy and hydrophilicity. Elnaz Ajami *et al.* also showed that the chemically modified hydrophilic implant surfaces exhibit faster and stronger bone formation (Ajami et al., 2021). Techniques such as sandblast and acid-etch (activated SLA) under nitrogen protection, UV, and non-thermal atmospheric plasma (NTAP) were proposed to get a hydrophilic surface on the implant without changing the surface roughness (Pesce et al., 2020). However, storing in a preservation solution such as sodium chloride might lead to time-dependent biological aging of the hydrophilic implant surface due to carbon contamination (Att and Ogawa, 2012; Henningsen et al., 2018a), which can affect the cell adhesion and overall osseointegration. Since hydrophilic treatment of dental implants is necessary by the chair side, new and efficient methods are warranted. Guo *et al.* have shown that 12 min of UV treatment or 1 min of NTAP treatment could make the implant surface hydrophilic and increase the adhesion of osteoblasts. This shortening of the processing time makes the implant NTAP treatment apparatus more practical for clinical routines (Guo et al., 2020). NTAP treatment can produce a high level of reactive oxygen/nitrogen species (ROS/RNS), high-density level of -OH and -NH_2_ groups, and less carbon contamination on the titanium implant surface (Jemat et al., 2015; Lee et al., 2017; Guo et al., 2020). Additionally, NTAP can functionalize titanium implants effectively and reduce bacterial adhesion, thereby reducing the incidence of peri-implantitis (Jungbauer et al., 2022).

For more convenience in the clinical procedure, a novel NTAP apparatus dedicated to chair-side implant hydrophilic activation was developed (Dong et al., 2021). For this apparatus, dielectric barrier discharge was adopted where argon (3,000 sccm; standard cubic centimeter per minute) and oxygen (0.3%) were used as working gases to activate plasma. Argon produces high levels of ROS/RNS, and a slight amount of oxygen can increase oxygen-containing groups on the titanium implant surface, which can further enhance the antibacterial properties and hydrophilicity of these biomaterials. Since the water contact angle of the NTAP-functionalized implant was close to 0°, the wetting of the whole implant surface needed only 3 s after contact with water (Dong et al., 2021). Consistent with previous studies, carbon contamination of the NTAP-functionalized implant surface was remarkably reduced and animal experiments have shown the osseointegration this way was faster and stronger compared to that of the traditional SLA titanium implant (Zheng et al., 2020; Dong et al., 2021). Wallkamm et al. (2015) have shown that the widely used hydrophilic Straumann SLActive implants had a favorable outcome for at least 3 years in their daily dental practice setup study. Interestingly, SLActive implants are sandblasted with large-grit aluminum oxide and acid etched before they are rinsed under nitrogen protection to minimize carbon contamination. It is noteworthy that the water contact angle is also close to 0. Although previous studies have shown that NTAP-functionalized implants are superior to traditional hydrophobic implants in terms of osteogenic capacity, it is still unclear if the osteogenesis effect of NTAP-functionalized implants is superior to that of commercially available hydrophilic implants.

Therefore, our aim in this study was to investigate the osteogenic effects of NTAP-functionalized titanium implants compared to commercially available SLActive™ implants at early stages. For this, we examined cell morphology, adhesion, and differentiation on customized SLA implant surfaces (Wego, Shandong, China) that were treated with NTAP and compared them to the phenotypes on the untreated SLActive implants, which gave us the effectiveness of the NTAP apparatus. Then, we utilized animal models to compare osseointegration between NTAP-treated and Straumann SLActive implants. Radiographic and histological analyses of specimens (implants and surrounding bone tissue) were used to measure osseointegration.

## 2 Materials and methods

### 2.1 NTAP treatment of implants

SLA implants were treated by a novel NTAP apparatus (CPActive, Chengdu, China) that was manufactured in our lab and has been described in a previous study (Dong et al., 2021). Briefly, the implant was handled with sterile forceps and placed on the collet. The collet was tightened, and the treatment chamber was closed manually. A plasma source by dielectric barrier discharge of 20w–50w was generated using 3,000 sccm/min of argon and 0.3% oxygen as a plasma source. The treatment time was set to 30 s.

### 2.2 *In vitro* experiment

#### 2.2.1 Implant preparation

Cylindrical Ti implants were prepared for *in vitro* experiments (grade IV, 4.3 mm in diameter, 8 mm in length, thread pitch 0.5 mm, Wego, Shandong, China). Sandblasting with large grit and acid etching was applied to the implant surface (SLA implant). SLA implants with and without NTAP treatment served as experimental and control groups, respectively, for the *in vitro* experiments.

### 2.2.2 Cell culture

MC3T3-E1 mouse preosteoblasts were offered by the State Key Laboratory of Oral Diseases (Sichuan University, China). Cells were maintained in the α-minimum essential medium (α-MEM, Gibco, Gaithersburg, MD, United States), containing 10% fetal bovine serum (FBS, Gibco, Gaithersburg, MD, United States) and 1% penicillin/streptomycin (PS, HyClone, Logan, UT, United States). The cells were cultured in an atmosphere of 5% CO_2_ at 37°C. The medium was refreshed every 3 days.

### 2.2.3 Cell morphology and attachment

MC3T3-E1 cells were seeded at a density of 1×10^4^ cells/well in 48-well plates with each well containing an implant. After 24 h, implants from the wells were taken out and fixed for 4 h in 4% paraformaldehyde. Fixed implants were washed with PBS three times, and specimens were then dehydrated in graded ethanol (50%, 75%, 90%, 95%, and 100%) and dried. The implants with adhered cells were observed by scanning electron microscopy (SEM) as described elsewhere (da Cruz et al., 2019). To evaluate early cell attachment, the implants were transferred into new wells at 6 h and 24 h of culture. Non-adherent cells were removed by washing the samples with PBS three times. The samples were fixed in 4% paraformaldehyde (PFA; Biosharp) for 20 min at room temperature (RT) and washed again with PBS before staining. The implants were stained with crystal violet solution (0.1%; Sigma-Aldrich) for 30 min at the RT (Parisi et al., 2019; Parisi et al., 2021) and washed with deionized H_2_O to remove excessive crystal violet stain. The bounded dye was solubilized using a 10% (volume/volume) acetic acid (Sigma-Aldrich), and the absorbance for the dye was measured at 620 nm using a microplate reader (Thermo Scientific).

### 2.2.4 Cell differentiation

The expression of osteogenic differentiation-related genes was detected by real-time polymerase chain reaction analysis (RT-qPCR) (Ma et al., 2018; Zheng et al., 2020). Briefly, MC3T3-E1 cells were seeded at a density of 1×10^4^ cells/well in 48-well plates, containing one implant per well. After 7 days and 14 days, implants with adhered cells were washed with PBS. Then, total RNA was extracted from adhered cells by using TRIzol reagent (Takara, Japan), and cDNA was synthesized using PrimeScript™ RT Master Mix (Takara, Tokyo, Japan) according to the manufacturer’s protocols. The transcription levels were determined by RT-qPCR with the SYBR Green PCR kit (Takara, Tokyo, Japan). Arithmetic formulae (2-ΔΔCt method) were applied to determine relative changes in gene expression over the internal control, β-actin. Primers used for RT-qPCR are as follows: β-actin (AGA​TTA​CTG​CTC​TGG​CTC​CTA​GC and ACT​CAT​CGT​ACT​CCT​GCT​TGC​T), osteogenic markers ALP (CTG​CCT​GAA​ACA​GAA​AGT​CTG​C and TAT​GTC​TTT​ACC​AGG​AGG​CGT​G), osteocalcin Ocn (GGA​CCA​TCT​TTC​TGC​TCA​CTC​TG and ACC​TTA​TTG​CCC​TCC​TGC​TTG), osteopontin Opn (TTC​TCC​TGG​CTG​AAT​TCT​GAG​G and GCT​GCC​AGA​ATC​AGT​CAC​TTT​C), and Runt-related transcription factor 2 Runx2 (ACG​AAA​AAT​TAA​CGC​CAG​TCG​G and CAC​TTC​ACC​CTC​AGG​ACC​G).

### 2.3 *In vivo* animal experiment

#### 2.3.1 Experimentation animals

Six healthy beagle dogs of ages ranging from 8 months to 1 year were included in the *in vivo* experiment. All dogs were provided by the Beagle Breeding Center, Sichuan Institute of Musk Deer Breeding, and housed in the West China Animal Room, Sichuan University (Chengdu, China). The experiment started after an adaptation period of 2 weeks. The dogs were housed individually and fed according to “Animal management regulations of China.” Free access to water was provided. All animal experiments have been approved by the ethics committee of Sichuan University (WCHSIRB-D-2021–003).

#### 2.3.2 Tooth extraction

For all surgeries in this study, animals were administered general anesthesia containing 0.04 ml/kg xylazole (Sumianxin II) and Zoletil (0.3 mg/kg) (Rong et al., 2018). The second to fourth premolars of the bilateral mandible were planned to be extracted (Manresa et al., 2014). After a high-speed turbine separated the crown, the mesial roots and the distal roots were extracted in stages with extraction forceps. Nylon thread was used for sutures.

#### 2.3.3 Implant surgical procedure

All implants for *in vivo* experiments (Straumann SLA and SLActive implants; bone level, 3.3*8 mm) were provided by the Straumann company (Switzerland). After 3 months of healing, implant surgery was performed according to the manufacturer’s instructions. Briefly, the surgical area was disinfected with iodophor after the animals were anesthetized. Three planting nests were prepared in each mandibular premolar area. Treatments were based on implant types and were divided into three groups (group Ⅰ: Straumann SLActive implant of 3.3*8 mm; group Ⅱ: Straumann SLA implant of 3.3*8 mm treated by NTAP; group Ⅲ: Straumann SLA implant of 3.3*8 mm). The implants were placed randomly in three planting nests by the same experienced implant surgeon (three implants per side). The distance between implants was maintained at at least 3 mm, and the distance between implants and natural teeth was maintained at at least 1.5 mm in our study. The shoulder of the implant was flushed with the alveolar ridge. For all implant installations, the insertion torque was stabilized at 35 Ncm. Cover screws were placed, and the flap was sutured. All animals were injected with penicillin (80,000 U) and dexamethasone (0.1 mg/kg) each day for 4 days after surgery (Rong et al., 2018). Painkillers (meloxicam tablets 0.1 mg/kg) were also provided with food to keep the animals comfortable.

#### 2.3.4 Specimen collection

The animals were observed for 2 w, 4 w, and 8 w, and two dogs were sacrificed at each time point, respectively, by intravenous injection of high-concentration potassium chloride. Specimens (bone tissue with implants) were collected and fixed in 4% paraformaldehyde. After 48 h, the specimens were transferred to 70% alcohol for storage (Wallkamm et al., 2015).

#### 2.3.5 Micro-computed tomography analysis

All specimens were scanned (specimens were placed in the scanning tube along the axis of implants) and analyzed by a micro-CT50 scanning system (Scanco Medical AG, Basserdorf, Switzerland) with a high resolution (Rong et al., 2018; Sanz-Esporrin et al., 2021). The X-ray source was set at 90 Kv and 200 μA with a voxel size of 15 μm. Once scanned, the implants with surrounding bone tissue were reconstructed in three dimensions. The circumferential region 500 μm away from the implant surface along the middle of the implant (4 mm in length) was defined as the volume of interest (VOI). Then, data were analyzed using SCANCO Medical Evaluation software. To differentiate implants and bone tissues, the gray threshold value range of the bone tissue was set up at 95–200 and the threshold value range of the implant was set up at 201–1,000. The investigator was blinded to specific groups. The bone parameters measured included 1) bone-to-implant contact (BIC; %), 2) bone volume fraction (BV/TV), 3) trabecular number (Tb.N), 4) trabecular thickness (Tb.Th), and 5) trabecular separation (Tb.Sp).

#### 2.3.6 Sectioning, staining, and analysis

**FIGURE 1:**
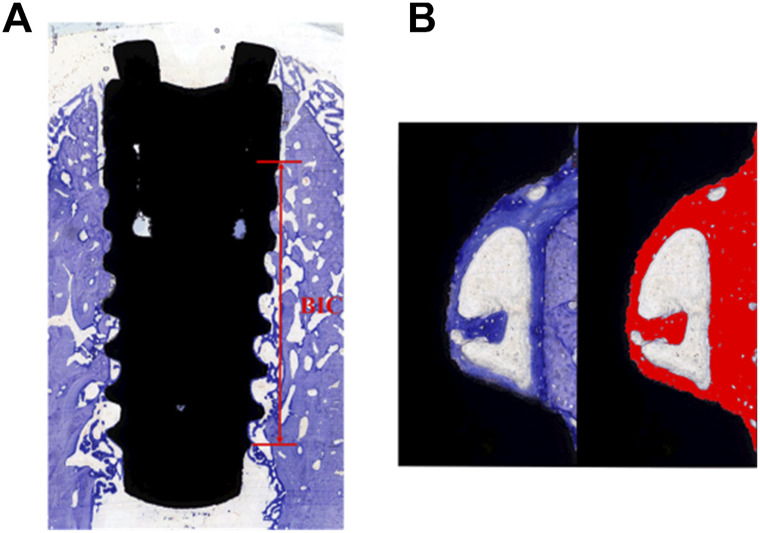
**(A)** Selected area of BIC% analysis. **(B)** Wound chamber tissue area assessment.

The specimens were sequentially dehydrated using 80%, 85%, 90%, 95%, and 100% ethanol and embedded in epoxy resin for 24 h (Rong et al., 2018). Dehydrated specimens were sliced in the buccolingual direction with a thickness of 100 μm using an E300CP diamond saw microtome (EXAKT, Germany). Hard-tissue sections were ground with the EXAKT 400 CS microchip grinder (about 50 μm thickness) and polished to a thickness of 30 um. After washing, the hard-tissue sections were stained with the toluidine blue solution according to the manufacturer’s instructions (Rong et al., 2018). The images were collected by CaseViewer (Panoramic MIDI; 3D HISTECH; Hungary). Analysis of the histological section was performed using ImageJ software. According to the description by Javier Sanz-Esporrin *et al.* before with minor changes, the percentage of histological BIC was calculated along a selected surface on the buccal and lingual aspects (Sanz-Esporrin et al., 2021). Histological analysis was used to determine the bone-to-implant contact percentage as BIC% = length of implant surface line with bone contact/total length of implant surface line (from first wound chambers to the last) (Figure 1A). Three wound chambers (on the middle of the implant) on each buccal and lingual aspect were selected to calculate the percentage of bone tissue (including newly formed bone and parent bone) considering the total wound chamber area as 100% (Figure 1B). Again, the investigator was blinded to the specific group division.

#### 2.3.7 Statistical analyses

All data were analyzed by IBM SPSS 26 and were presented as means ± standard deviation. Student’s t test was used to assess crystal violet staining and RT-qPCR results in an *in vitro* experiment. Non-parametric statistics for paired data (Kruskal–Wallis test) with the Bonferroni correction was used for analyzing significant differences in micro-CT data. The Friedman test was used to analyze significant differences in histological data. For all analyses, *p*-value <0.05 was considered significant.

## 3 Results

### 3.1 *In vitro* experiment

#### 3.1.1 NTAP treatment enhanced cell initial attachment and differentiation

**FIGURE 2:**
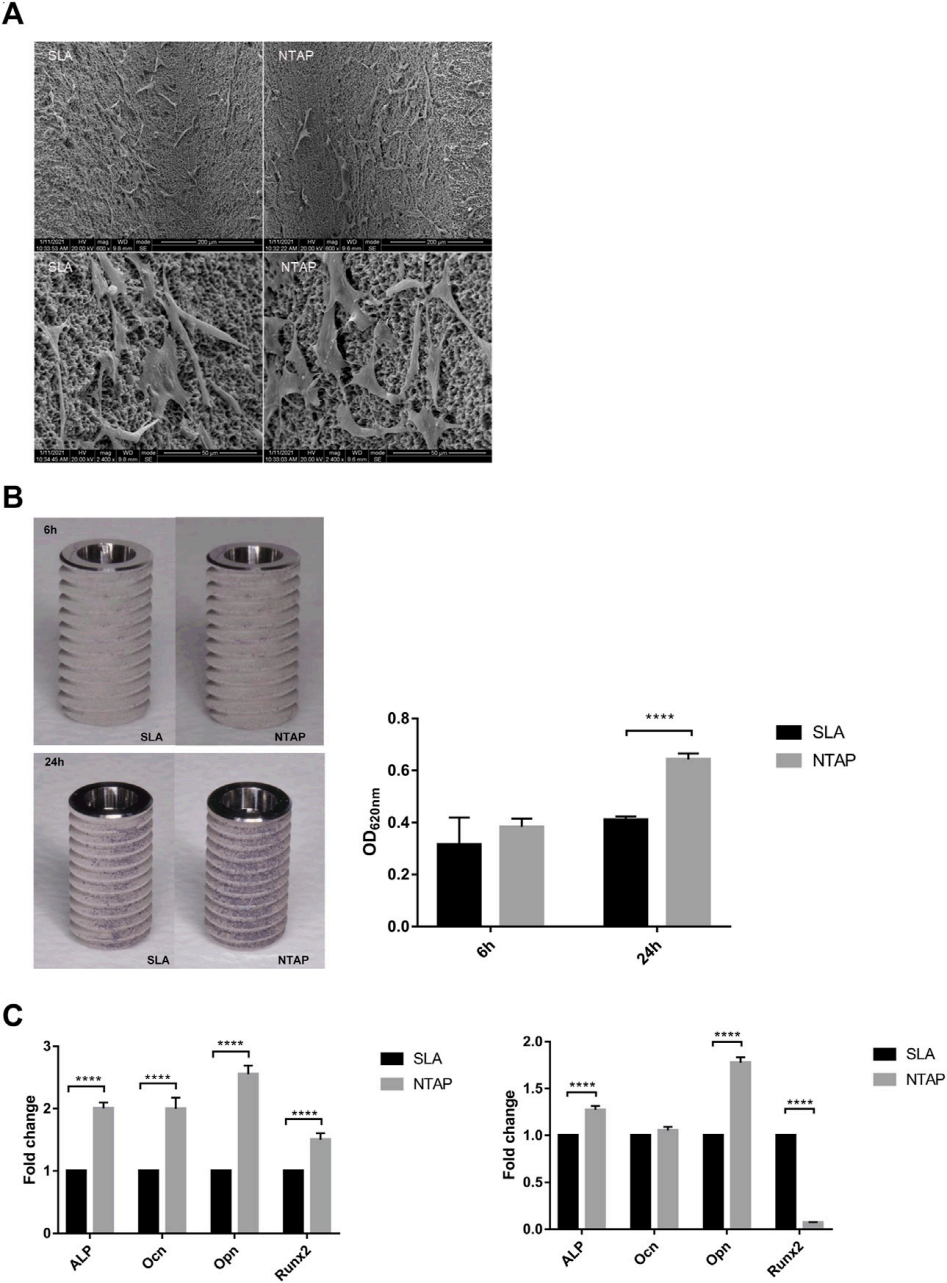
Biological behavior of MC3T3-E1 cells on NTAP and SLA implants. **(A)** Cell morphology at 12 h observed by SEM (magnification: ×600 and ×2400). **(B)** Cell adhesion at 6 h and 24 h measured by crystal violet staining. **(C)** Expression of osteogenic differentiation-related genes (*alp*, *ocn*, *opn*, and *runx2*) of MC3T3-E1 cells at 7 days (left) and 14 days (right) tested by RT-qPCR. Values represent the means of data obtained from three different experiments with standard deviations. **** = *p* < 0.001 of Student’s t test using IBM SPSS 26 (the error bars indicate standard deviations).

As shown in Figure 2A, SEM analysis indicated that cells on the NTAP-functionalized titanium implant surface displayed a great stretch with more protrusions compared to those in the control group. Crystal violet staining was used to account for the number of cells adhered to the two implant surfaces. Up to 30% more cells adhered to the NTAP-functionalized implant surface than to the control group after 24 h of attachment (*p* < 0.05) (Figure 2B). Furthermore, the expression of osteogenic differentiation genes (*alp*, *ocn*, *opn*, and *runx2*) was analyzed by RT-qPCR. By day 7 of culture, cells on the NTAP-functionalized surface had significantly higher levels of *alp*, *ocn*, *opn*, and *runx2* expression than the cells cultured on the SLA surface (*p* < 0.05) (Figure 2C). However, after 14 days, only *alp* and *opn* were upregulated in the NTAP group. The expression of *runx2* in the NTAP group evidently decreased than that in the control group (Figure 2C).

### 3.2 *In vivo* experiment

#### 3.2.1 Micro-CT results

**FIGURE 3:**
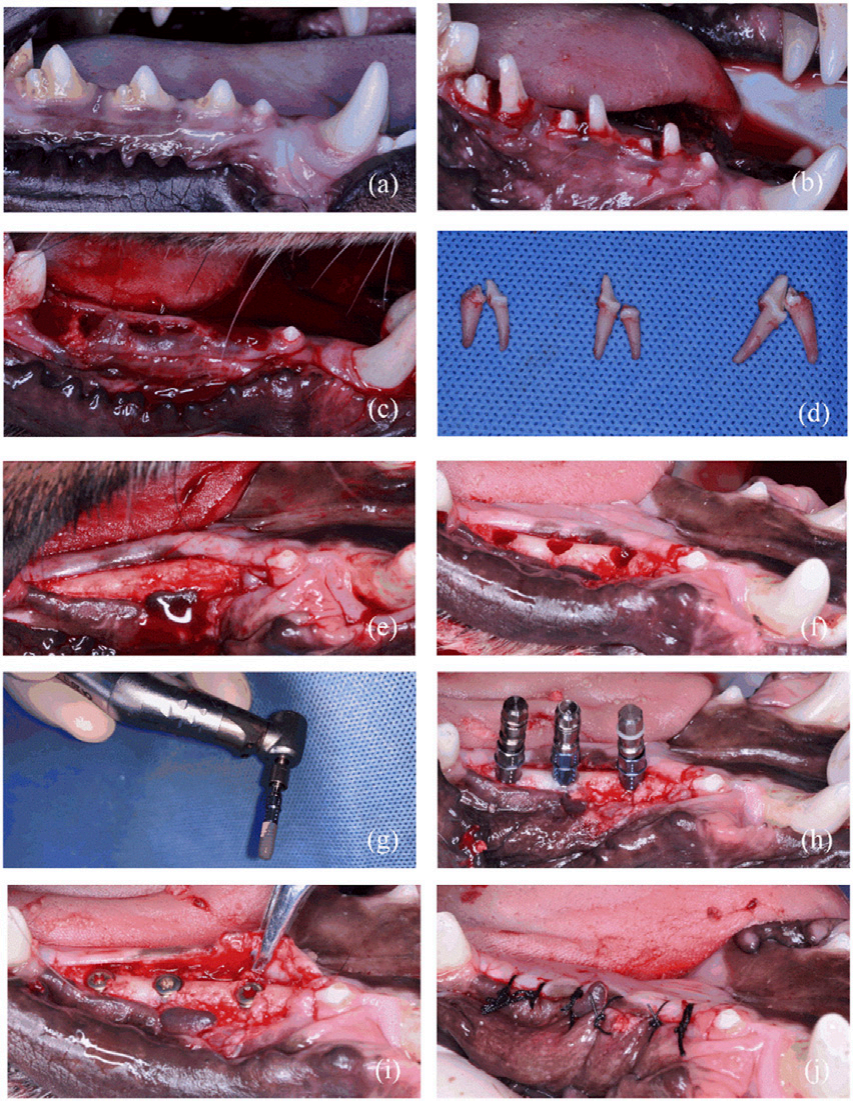
Surgical stages of *in vivo* experiment. **(A)** Preoperative photo. **(B)** Teeth hemisection prior to extraction. **(C)** Wound after extractions. **(D)** Isolated teeth. **(E)** Flap after 3-month healing of extractions. **(F)** Planting nest preparation. **(G)** Hydrophilic effect of implant treated with NTAP. **(H)** Implant placement. **(I)** Screw on the cover screw. **(J)** Suture after implant placement.

**FIGURE 4:**
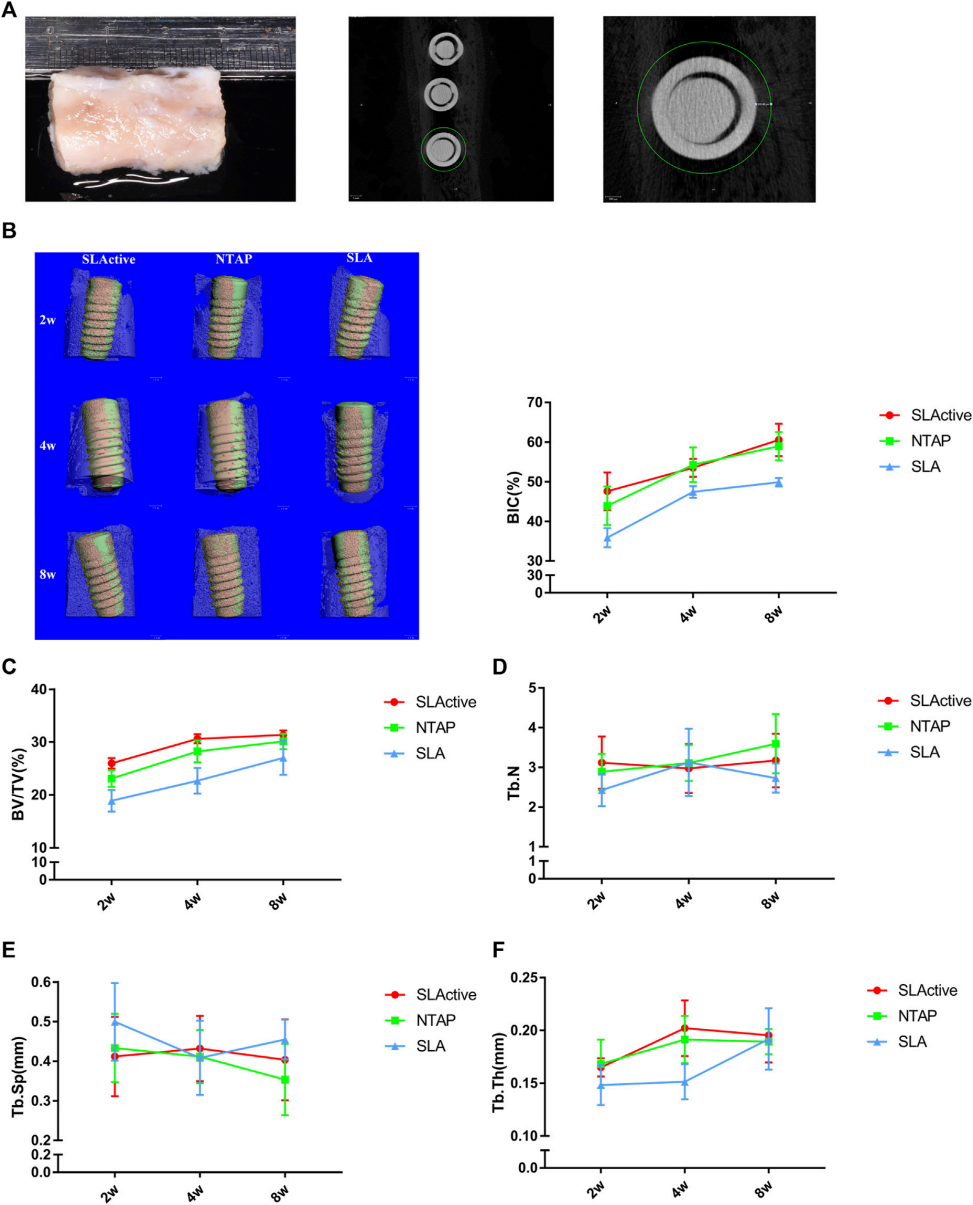
Micro-CT analysis of SLActive, NTAP, and SLA implants with the surrounding bone tissue (*n* = 4). **(A)** Mandible specimens with implants and transverse micro-CT view (left), and a region of interest (ROI) was defined as the circumferential region exactly 500 μm away from the implant surface. **(B)** BIC% of the implant at 2 w, 4 w, and 8 w (left: 3D reconstruction; orange represents the direct contact between the bone and implants). **(C)** Bone volume fraction (BV/TV). **(D)** Trabecular thickness (Tb.N). **(E)** Trabecular separation (Tb.Sp). **(F)** Trabecular thickness (Tb.Th). Values represent the means of data obtained from three different experiments with standard deviations. * = p < 0.05 of the Kruskal–Wallis test with the Bonferroni correction using IBM SPSS 26 (the error bars indicate standard deviations).

For the *in vivo* experiment, the surgical procedure is shown in Figure 3. The specimens were obtained and scanned with micro-CT. A volume of interest (VOI) of 4 mm in length was selected in the middle of the implant with the surrounding bone tissue for all specimens (Figure 4A). Radiographic bone-to-implant contact was analyzed at 2 w, 4 w, and 8 w. The results showed no statistical difference for the BIC% of the three groups observed at all time points. The mean BIC% in the NTAP group was slightly lower than that of the SLActive group at 2 w and 8 w (Figure 4B and Table 1). Additionally, the radiographic BIC% of the SLActive and NTAP groups was higher by 11.75% and 8.06% at 2 w, when compared with the SLA group. By week 4 of healing, the radiographic BIC% of the SLActive group was higher by 6.08% and that of the NTAP group was higher by 6.88% than that of the SLA group. Furthermore, after 8 weeks of healing, BIC% of the SLActive and NTAP groups demonstrated significant increases of 10.76% and 9.11% in contrast to the SLA group.

**TABLE 1:** Micro-CT radiographic BIC %, BV/TV value, Tb.N, Tb.Sp, and Tb.Th in the four middle mm of the implant surfaces in different groups: 2-week healing, 4-week healing, and 8-week healing in both implant groups.

	Group	2 w	4 w	8 w
BIC (%)	SLActive	47.605 ± 4.743	53.508 ± 2.246	60.558 ± 4.109
	NTAP	43.913 ± 4.883	54.303 ± 4.372	58.968 ± 3.549
	SLA	35.853 ± 2.401	47.423 ± 1.455	49.863 ± 1.043
	△SLActive—NTAP	1.000	1.000	1.000
	△SLActive—SLA	0.102	0.102	0.102
	△NTAP—SLA	0.102	0.102	0.102
BV/TV (%)	SLActive	25.980 ± 0.988	30.628 ± 0.844	31.363 ± 0.844
	NTAP	23.120 ± 1.584	28.255 ± 2.074	30.123 ± 1.468
	SLA	18.898 ± 2.036	22.688 ± 2.438	27.010 ± 3.213
	△SLActive—NTAP	0.472	1.000	-
	△SLActive—SLA	0.014^a^	0.040^a^	-
	△NTAP—SLA	0.472	0.231	-
Tb.N	SLActive	3.118 ± 0.658	2.971 ± 0.614	3.171 ± 0.676
	NTAP	2.888 ± 0.454	3.109 ± 0.453	3.593 ± 0.747
	SLA	2.428 ± 0.409	3.125 ± 0.848	2.729 ± 0.365
	△SLActive—NTAP	1.000	-	0.472
	△SLActive—SLA	0.040^a^	-	0.014^a^
	△NTAP—SLA	0.231	-	0.472
Tb.Sp (mm)	SLActive	0.412 ± 0.100	0.432 ± 0.083	0.404 ± 0.102
	NTAP	0.433 ± 0.086	0.412 ± 0.067	0.354 ± 0.090
	SLA	0.500 ± 0.098	0.408 ± 0.094	0.455 ± 0.050
	△SLActive - NTAP	1.000	-	0.472
	△SLActive - SLA	0.040^a^	-	0.014^a^
	△NTAP - SLA	0.231	-	0.472
Tb.Th (mm)	SLActive	0.165 ± 0.009	0.202 ± 0.026	0.195 ± 0.026
	NTAP	0.168 ± 0.023	0.191 ± 0.022	0.189 ± 0.012
	SLA	0.148 ± 0.019	0.151 ± 0.017	0.192 ± 0.029
	△SLActive—NTAP	1.000	1.000	-
	△SLActive—SLA	0.102	0.040^a^	-
	△NTAP—SLA	0.102	0.231	-

^a^ Comparisons between groups. *p* < 0.05. *p*-value in the tables was obtained by the Kruskal–Wallis test with the Bonferroni correction.

^b^ No statistical difference among the three groups was observed.

To measure the trabecular bone parameters, a region of interest (ROI) was defined as a circumferential region exactly 500 μm away from the implant surface along the length as shown in Figure 4A. After a 2-week post-treatment, trabecular bone volume fraction (BV/TV) values were measured, and interestingly, the BV/TV value of SLActive implants was slightly higher than that of the NTAP group at 2, 4, and 8 weeks (*p* > 0.05). The BV/TV value difference between the SLActive and NTAP groups was not found to be significant. Furthermore, at the point of 2 and 4 weeks, the mean BV/TV values of the SLActive and NTAP groups demonstrated notable increases than those of the SLA group (Figure 4C and Table 1).

To observe the difference in the trabecular bone around the implant, the number (Tb.N), separation (Tb.Sp), and thickness (Tb.Th) were also examined. Compared to the SLA group, the Tb.N value of the SLActive group demonstrated a significant increase at 2 and 8 weeks (*p* < 0.05). There was no significant difference between the NTAP group and SLActive groups (Figure 3D and Table 1). Moreover, Tb.Sp of the SLActive group showed a marked reduction at 2 weeks, and the NTAP group showed a reduction at both 2 and 8 weeks in contrast to the SLA group. There was no notable difference observed between the SLActive and NTAP groups (Figure 4E and Table 1). However, Tb.Th of the SLActive and NTAP groups was observed to be higher than that of the SLA group at 4 weeks (*p* > 0.05) (Figure 4F and Table1).

### 3.3 Toluidine blue staining results

**FIGURE 5:**
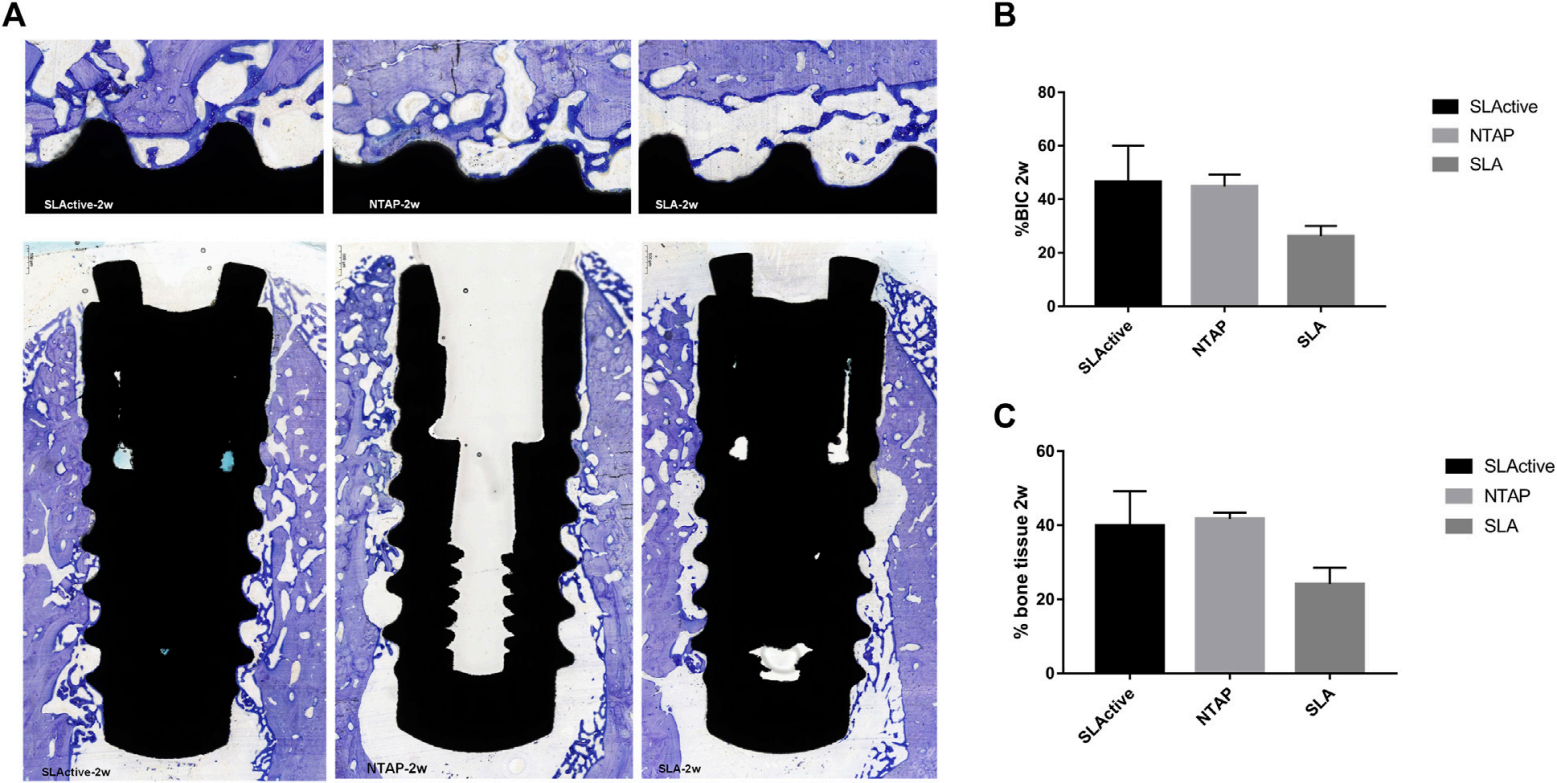
Histological section and analysis after 2 weeks of implantation (*n* = 3). **(A)** New bone formation was assessed by toluidine blue staining in the SLActive, NTAP, and SLA groups. Magnification: upper ×10 and lower ×2 and scalebar = 500 μm. **(B)** Percentage of histological BIC. **(C)** Percentage of bone tissue in wound chamber. Values represent the means of data obtained from three different experiments with standard deviations. * = *p* < 0.05 of the Friedman test using IBM SPSS 26 (the error bars indicate standard deviations).

Toluidine blue staining was carried out to identify newly formed bone from the parent bone with deeper staining for the newly formed bone as compared to the parent bone. It can be seen that the new bone grew from the parent bone and gradually extended to the wound chambers of the implant surface (Figure 5A). The ground sections showed that several wound chambers contained new bones in the SLActive and NTAP groups. However, new bone tissue could be observed only in a few wound chambers on the SLA surface (Figure 5A). Statistically, the mean percentage of histological BIC and bone tissue in the wound chambers of the three groups showed no difference. The mean percentage of histological BIC value and bone tissue in wound chambers in the SLActive and NTAP groups was similar, but the histological BIC value and bone tissue in wound chambers in the SLA group displayed a notable reduction compared with the SLActive and NTAP groups (Figure 5C and Table 2).

**TABLE 2:** Percentage of histological BIC and bone tissue in different groups: 2-week healing, 4-week healing, and 8-week healing in implant groups.

	Group	2 w	4 w	8 w
% Histological BIC	SLActive	46.488 ± 13.578	55.648 ± 5.440	67.848 ± 67.848
	NTAP	44.834 ± 4.419	54.088 ± 13.087	63.177 ± 3.621
	SLA	26.174 ± 3.843	38.315 ± 4.632	47.269 ± 5.694
	p-value	0.660	0.113	0.061
% Bone tissue	SLActive	39.861 ± 9.347	39.702 ± 2.330	45.532 ± 2.815
	NTAP	41.729 ± 1.703	41.209 ± 6.159	45.197 ± 2.504
	SLA	24.129 ± 4.452	30.749 ± 3.688	36.906 ± 3.624
	p-value	0.660	0. 660	0.660

*p*-value was obtained by the Friedman test. No statistical difference among the three groups was observed.

**FIGURE 6:**
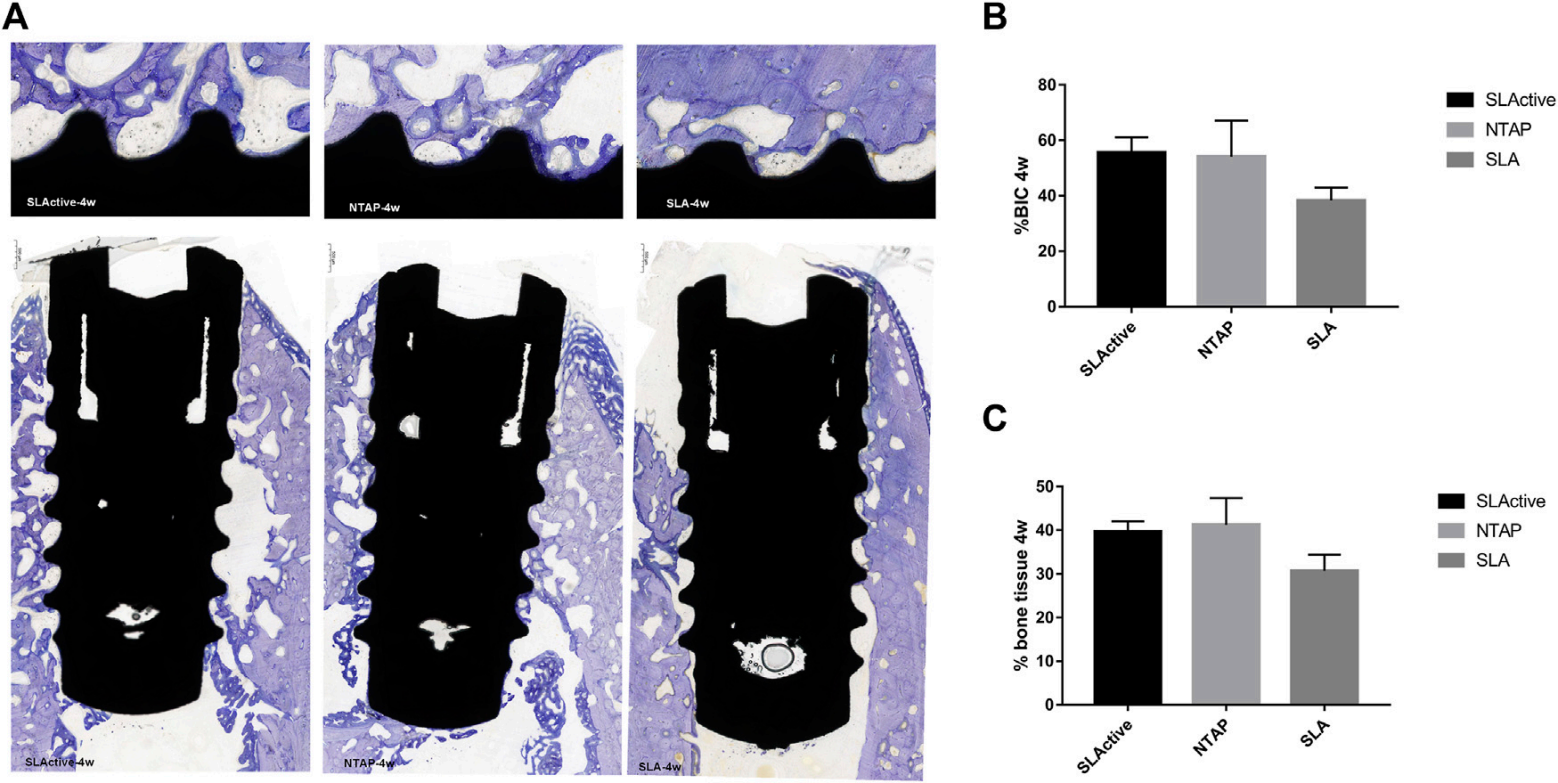
Histological section and analysis after 4 weeks of implantation (*n* = 3). **(A)** New bone formation was assessed by toluidine blue staining in the SLActive, NTAP, and SLA groups. Magnification: upper ×10; lower ×2 and scalebar = 500 μm. **(B)** Percentage of histological BIC. **(C)** Percentage of bone tissue in wound chamber. Values represent the means of data obtained from three different experiments with standard deviations. * = *p* < 0.05 of the Friedman test using IBM SPSS 26 (the error bars indicate standard deviations).

By week 4 of healing, as the regenerated trabecular bone gradually matured, its staining was less distinguishable than in the new bone at week 2. The wound chambers of the implant surface contained a higher amount of new bone in the SLActive and NTAP groups. However, a thin layer of new bone could be observed on the SLA surface (Figure 6A). Although the difference among the three groups was not statistically significant, the mean percentage of histological BIC and bone tissue of the SLActive and NTAP groups was higher than that of the SLA group (Figures 6B, C; table 2). The SLActive and NTAP groups still displayed similar means of histological BIC% and bone tissue in wound chambers.

**FIGURE 7:**
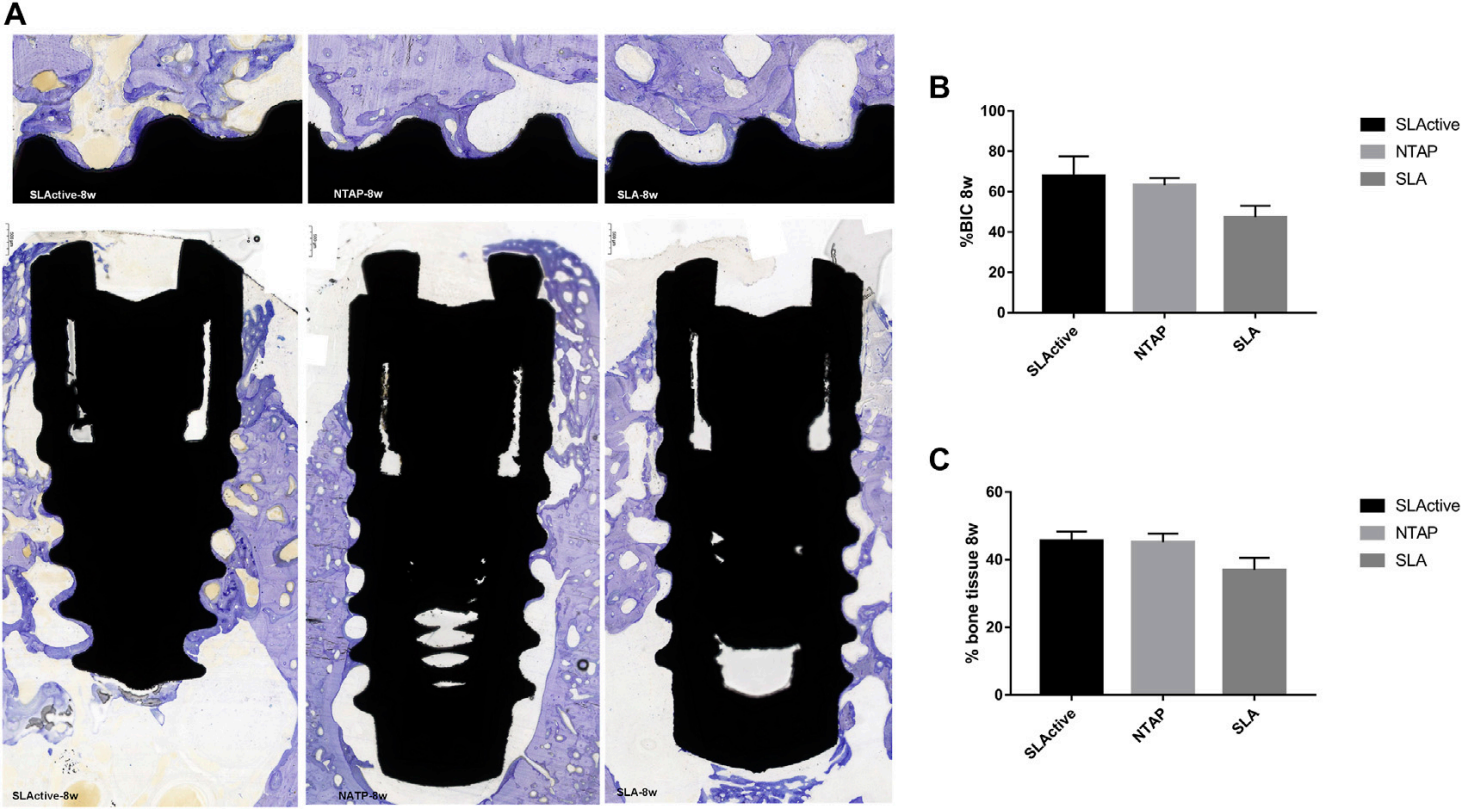
Histological section and analysis after 8 weeks of implantation (*n* = 3). **(A)** New bone formation was assessed by toluidine blue staining in the SLActive, NTAP, and SLA groups. Magnification: upper ×10; lower ×2 and scalebar = 500 μm. **(B)** Percentage of histological BIC. **(C)** Percentage of bone tissue in wound chamber. Values represent the means of data obtained from three different experiments with standard deviations. * = *p* < 0.05 of the Friedman test using IBM SPSS 26 (the error bars indicate standard deviations).

After 8 weeks of implantation, the regenerated bone tissue was more mature and the staining became lighter. The wound chambers of the implant surface were occupied heavily with new bone in three groups (Figure 7A). Although there was no significant difference, the means of histological BIC% in the NTAP group were higher than those in the control (Figure 7B and Table 2). Moreover, percentages of bone tissue in wound chambers in the SLActive and NTAP groups were higher than those of the SLA group (*p* > 0.05) (Figure 7C and Table 2) (*p* < 0.05). For new bone formation, the NATP group and the SLActive group demonstrated similar effects.

## 4 Discussion

Many methods have been proposed and attempted to modify the implant surface to enhance the biocompatibility of titanium implants and increase the bone-to-implant contact without changing the surface morphology and roughness. Increasing the hydrophilicity of the titanium implant surface proved to be a feasible way. Compared with a hydrophobic surface, a hydrophilic surface with high surface energy was favorable to osteoblast adhesion and subsequently new bone formation (Hong et al., 2013; Jinno et al., 2021). However, the biological aging of titanium surfaces is inevitable due to long storage and carbon contamination (Att and Ogawa, 2012). Hellen S. Teixeira et al. proposed that the hydrophilic treatment of the implant by the chair-side immediately prior to its placement minimized carbon contamination and maintained its high surface energy. UV and NATP functionalization could both increase surface energy and get a hydrophilic implant surface. Canullo L *et al.* found osteoblasts treated with 12 min argon-based plasma or 3 h UV light displayed similar cell adhesion and surface protein adsorption (Canullo et al., 2016; Henningsen et al., 2018b). Considering the convenience and efficiency of the clinical procedure, NATP generators were regarded as potential candidates for surface hydrophilic activation by the chair side.

We previously generated an argon- and oxygen-based NTAP implant hydrophilic apparatus (CPActive, Chengdu, China). Argon and a slight amount of oxygen (0.3%) were used for generating plasma, and treatment for 30 s with this apparatus made a super-hydrophilic titanium implant surface. Compared to the oxygen plasma generator (Diener Electronic GmbH, Ebhausen, Germany; gas flow rate of 1.25 sccm, and gas purity of >99.5%), the processing time of argon- and oxygen-based NTAP was reduced by half (Guo et al., 2020). Implant surfaces have been previously cleaned with argon plasma to remove contaminants and other impurities (Aronsson et al., 1997). Our previous studies have shown that after treating the titanium implant with this apparatus for 30 s, it significantly reduces the carbon element on the titanium surface (Zheng et al., 2020; Dong et al., 2021). A slight amount of oxygen (0.3%) plasma efficiently kills bacteria (Annunziata et al., 2016). In addition, argon and oxygen plasma could increase the number of free radicals (mainly -OH) and surface energy level on the titanium surface. Zheng et al. (2020) demonstrated argon- and oxygen-based NTAP-treated Ti disks enhanced MC3T3-E1 cell attachment and osteogenic differentiation. Furthermore, in this way, there was a significant upregulation of osteogenic differentiation genes (*alp*, *ocn*, *opn*, and *runx2*) (Zheng et al., 2020). In order to test the effect of argon- and oxygen-based generators on the implant with threads, the morphology of MC3T3-E1 cells, their initial attachment, and differentiation on an NTAP-treated implant surface were determined. Consistent with the research of Lei Wang et al. (2020), osteoblasts were fibroid or polygonal with thick and long synapses on NTAP-treated SLA titanium discs, whereas cells on hydrophobic SLA titanium discs were shrunken and in a round shape. On the other hand, NTAP treatment promoted cell initial adhesion and osteogenic differentiation, especially at the early stages. Upregulation of *alp*, *ocn*, and *opn* indicated an increase in alkaline phosphatase activity, calcification, and osteogenic marker levels (Yang et al., 2019). The hydrophilic surface promoted protein adsorption and altered the integrin-mediated signaling pathways (Wang et al., 2020).

Our results were consistent with those of Natalie R. Danna *et al.*, who reported that BIC% of titanium implants treated with a plasma jet (INP, Greifswald, Germany; 16% oxygen, 1% hydrogen, and 78% nitrogen) in animal models was >10% on average than that of implants without NTAP treatment at 6 weeks (Danna et al., 2015). In our study, micro-CT analysis showed the BIC% of the NTAP and SLActive groups was higher than that of the SLA group at early osseointegration. Furthermore, there was no statistically significant difference between the SLActive and NTAP groups, though the mean BIC% in the NTAP group was slightly lower than that of the SLActive group at 2 w and 8 w. The BV/TV value directly reflects changes in bone mass. BV/TV of the SLActive and NTAP groups were notably enhanced at 2 w and 4 w. BV/TV of the SLActive group was comparable to that of the NTAP group, indicating both SLActive and NTAP implants presented similar ability of osteogenesis, especially in the early stage. For trabecular bone parameters, Tb.Th demonstrated minimal difference among the three groups. It can be speculated that the wettability of the implant surface had a minor effect on trabecular thickness. An increase in hydrophilicity and biological activity enhanced bone mass, trabecular number, and density, which were beneficial for the long-term stability of implants in the jaw.

In addition to the initial stability of implant anchoring in the jaw, secondary stability is crucial for a successful implant restoration, which mainly depends on new bone formation (Ghanavati et al., 2006; Guglielmotti et al., 2019). Analysis of toluidine blue staining of histological sections clearly indicated regenerated bone. The new bone grew from the original bone and gradually extended to the wound chambers of the implant surfaces, and this performance was consistent with an implant sequential healing study by Riccardo Favero et al. (2016). The new bone gradually matured after implantation and the boundary between the new bone and the old bone changed from being clear to fuzzy from 2 w to 8 w. Furthermore, the mean histological BIC% and bone tissue in the wound chambers in the SLActive and NTAP groups were higher than those in the SLA group at all time points, though no statistical difference could be observed. Either way, it was clear that NTAP treatment enhanced implant early osseointegration. The lack of statistical difference may be due to the inability to perform paired tests (it was difficult to get complete hard-tissue sections from all specimens) and a small sample experiment. The mean percentage of histological BIC value and bone tissue in the SLActive and NTAP groups were similar, indicating that NTAP treatment-enhanced osseointegration and its osteogenesis effect were comparable to those of the Straumann SLActive implants histologically.

Taken together, NTAP-functionalized and SLActive titanium implant surfaces enhanced new bone formation at the early stage. Moreover, the osteogenesis effects of implants treated with NTAP chair-side were comparable to those of the Straumann SLActive implant radiographically. When we have not demonstrated a significant difference among the three implant surfaces on histological BIC and bone tissue in wound chambers, future experiments with a larger sample size are warranted. Considering different bone remodeling between humans and animals, clinical studies on humans should be considered.

## Data Availability

The original contributions presented in the study are included in the article/Supplementary Materials; further inquiries can be directed to the corresponding author.
